# From exercise to emergency—an extreme presentation of MINOCA: a case report

**DOI:** 10.1093/ehjcr/ytag442

**Published:** 2026-06-11

**Authors:** Aswin Babu, Ciara Kennedy, Travis Chong, Jon Spiro

**Affiliations:** Cardiology Department, Royal Perth Hospital, Victoria Square, Perth, Western Australia 6000, Australia; Medical School, University of Western Australia, Crawley, Western Australia 6009, Australia; Cardiology Department, Royal Perth Hospital, Victoria Square, Perth, Western Australia 6000, Australia; Cardiology Department, Royal Perth Hospital, Victoria Square, Perth, Western Australia 6000, Australia; Cardiology Department, Royal Perth Hospital, Victoria Square, Perth, Western Australia 6000, Australia; Medical School, University of Western Australia, Crawley, Western Australia 6009, Australia

**Keywords:** Case reportc Coronary artery vasospasm, CAS, Myocardial infarction with non-obstructive coronary arteries, MINOCA, Out of hospital cardiac arrest, OOHCA Coronary function testing, CFT

## Abstract

**Background:**

Coronary artery vasospasm (CAS) is an important, yet under-recognized cause of myocardial infarction with non-obstructive coronary arteries (MINOCA), associated with significant morbidity and mortality.

**Case summary:**

A 51-year-old male presented with an out of hospital ventricular fibrillation (VF) arrest. Coronary angiography with intravascular imaging revealed unobstructed epicardial coronary arteries and cardiac magnetic resonance imaging (CMRi) demonstrated a structurally normal heart with no evidence of fibrosis or myocardial oedema. High-sensitivity troponin I was elevated, confirming acute myocardial injury consistent with a working diagnosis of MINOCA. Subsequent comprehensive coronary function testing (CFT) demonstrated significant spasm fulfilling all three COVADIS diagnostic criteria for definitive epicardial CAS, and timely endotype specific medical therapy was instituted.

**Discussion:**

This case highlights the importance of considering CAS in unexplained cardiac arrest. Specific disease endotyping allows a stratified and personalized medical treatment regimen to enhance clinical outcomes and prevent recurrences.

Learning points

- MINOCA should be regarded as a working diagnosis requiring systematic evaluation including coronary function testing.
- Coronary artery vasospasm (CAS) is an under-recognized cause of out-of-hospital cardiac arrest that mandates endotype-specific therapy.
- ICD implantation should be considered in CAS-related cardiac arrest survivors following shared decision-making.

## Introduction

Myocardial infarction with non-obstructive coronary arteries (MINOCA) represents a heterogeneous group of conditions that account for 6–10% of all acute myocardial infarctions.^1,2^ The 2023 European Society of Cardiology (ESC) guidelines for the management of acute coronary syndromes formally established MINOCA as a working diagnosis that mandates systematic evaluation to identify the underlying cause.^3^ Of these, coronary artery vasospasm (CAS) is an important and potentially life-threatening condition that is often under-recognized.^4,5^ Furthermore, the 2024 ESC chronic coronary syndromes guidelines have upgraded invasive coronary function testing (CFT) to a Class I (Level B) recommendation,^6^ and both Japanese and American consensus documents endorse its use in unexplained cardiac arrest.^7,8^ Yet, despite guideline recommendations, CFT remains underutilized and the diagnosis of CAS is often delayed or missed.^8,9^

In the following case report, we describe a previously fit and well middle-aged man who presents with an out of hospital cardiac arrest (OOHCA) secondary to CAS, illustrating the importance of comprehensive CFT to enable precise endotype characterization for targeted medical therapy.

## Summary figure

**Figure:**
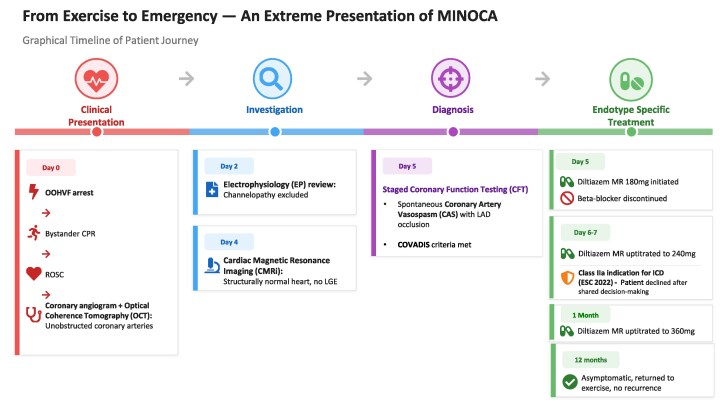


## Case presentation

**Figure 1:**
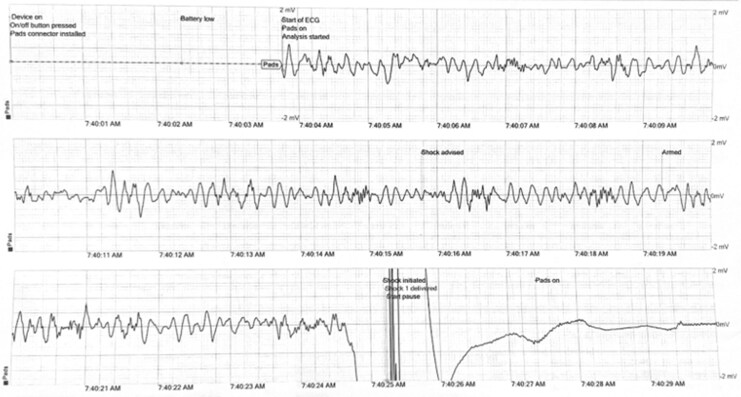
Electrocardiogram from emergency services demonstrating ventricular fibrillation and delivery of shock.

**Figure 2:**
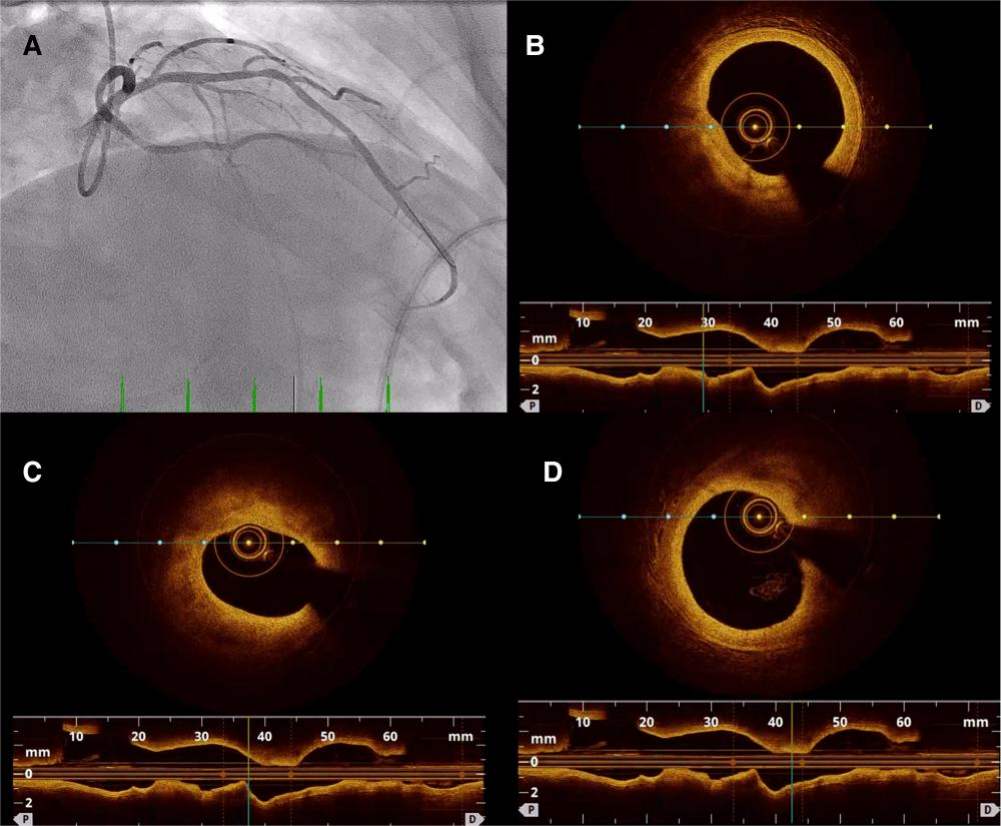
(*A*) Angiographic still frame of the LAD at index procedure demonstrating unobstructed coronaries with mild mid-LAD disease; (B–D) representative OCT cross-sections excluding plaque rupture, erosion, SCAD, and thrombus.

A 51-year-old gentleman suffered an out of hospital ventricular fibrillation (OOHVF) arrest whilst exercising at the gym (*Figure 1*). Immediately beforehand, he developed chest pain with radiation to the left arm. Bystander CPR was promptly initiated, with a single automated external defibrillator (AED) shock delivered to achieve return of spontaneous circulation (ROSC). Post-ROSC electrocardiogram (ECG) revealed anterior ST elevation myocardial infarction (STEMI) and the patient was transferred directly to the cardiac catheterization laboratory. His past medical history was pertinent for recently diagnosed hypertension, but he was medication naïve. Further, he was an ex-smoker with a 10-year pack history who currently vapes. Coronary angiogram demonstrated unobstructed coronary arteries with only mild disease within the mid-left anterior descending artery (LAD). Optical coherence tomography (OCT) confirmed the absence of intravascular thrombus, spontaneous coronary artery dissection (SCAD), plaque rupture, or erosion (*Figure 2*, Supplementary material online, *Video S1*).

To further define the aetiology, a systematic evaluation was undertaken. Cardiac magnetic resonance imaging (CMRi) confirmed a structurally normal heart with preserved biventricular function, no myocardial oedema and no late gadolinium enhancement (see Supplementary material online, *Video S2A*, *S2B*, *S2C*). Notably, the absence of apical or mid-ventricular ballooning on CMRi made Takotsubo cardiomyopathy unlikely. An exercise stress ECG was normal and electrophysiology (EP) review excluded an underlying channelopathy. High-sensitivity troponin I was elevated at presentation (4 ng/L; <26 ng/L), peaking at 77 ng/L at 24 h, supporting acute myocardial injury. On the basis of a dynamic rise in tr1oponin, anterior ST elevation and unobstructed coronary arteries, a working diagnosis of MINOCA complicated by cardiac arrest was established.^3^

**Figure 3:**
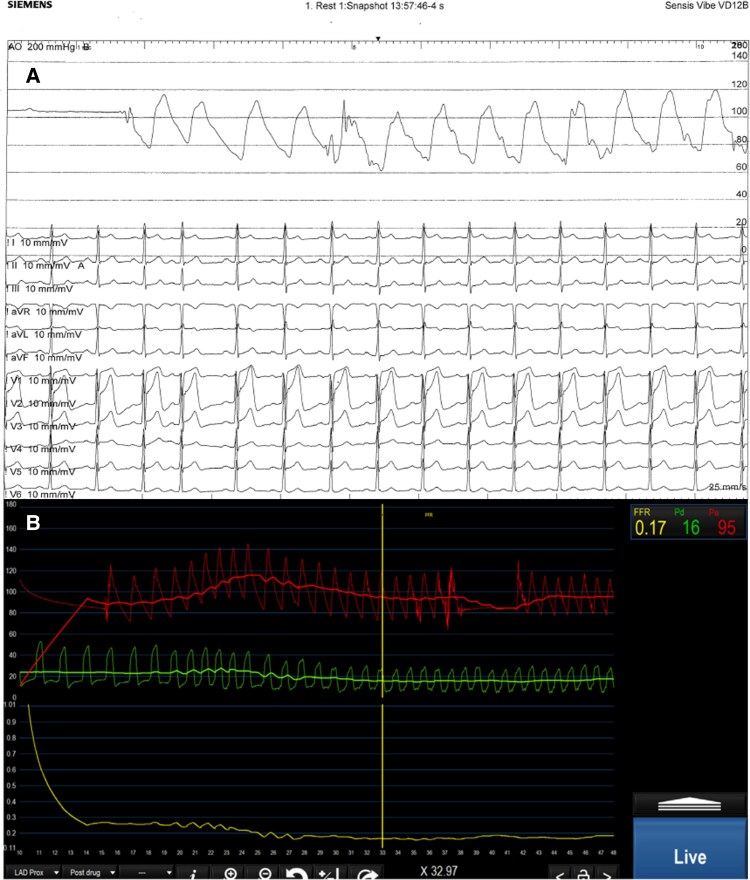
(*A*) A 12-lead electrocardiogram exhibiting ST elevation in the anterior leads during coronary function testing (CFT). (*B*) Significant spontaneous drop in LAD Pd/Pa pressure tracing consistent with vessel occlusion.

**Figure 4:**
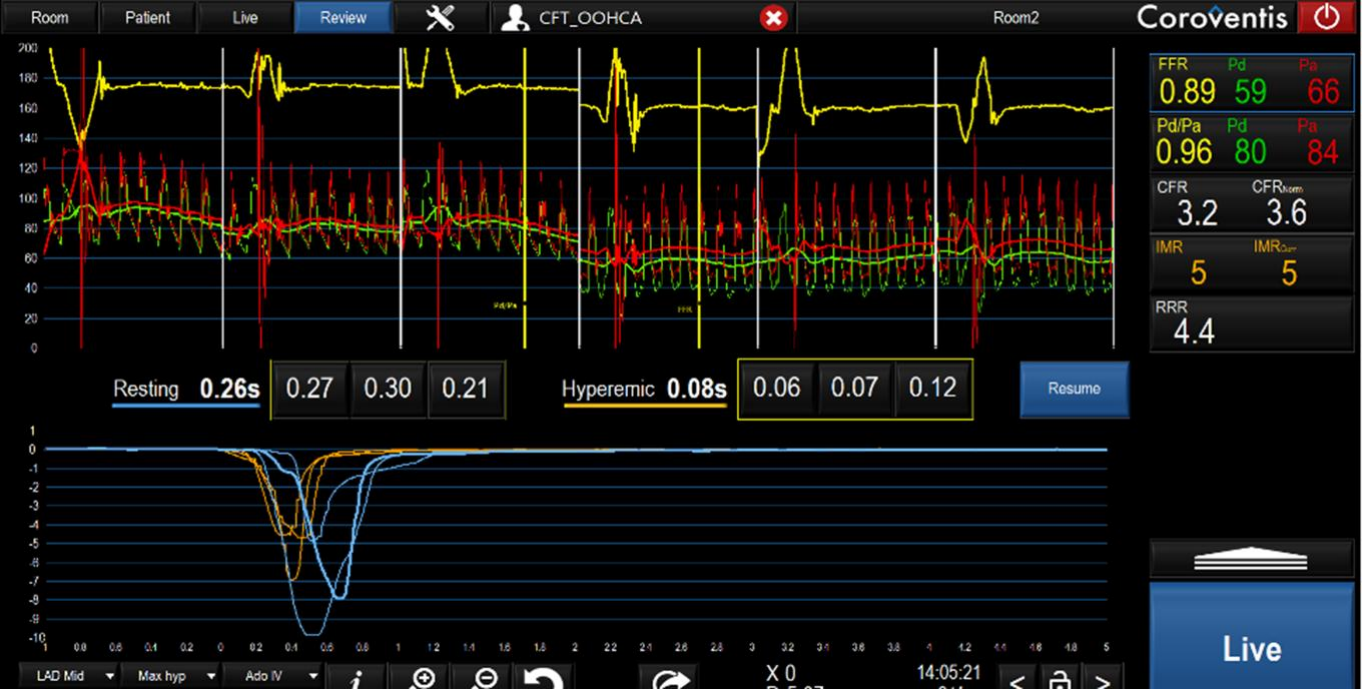
Coroventis screen demonstrating transit times and bolus thermodilution curves during rest and hyperaemia. Coronary flow reserve (CFR) = 3.2 and index of microcirculatory resistance (IMR) = 5, both within normal limits.

Given the clinical suspicion for CAS, a staged CFT procedure with planned acetylcholine provocation was performed. However, following passage of a pressure/thermistor guidewire (PressureWire X, Abbott) along the LAD, significant spontaneous CAS was demonstrated with complete closure of the mid-LAD (see Supplementary material online, *Video S3A*). This was associated with reproduction of presenting symptoms, anterior ST elevation, and a precipitous drop in Pd/Pa to 0.17 (*Figure 3*). The presentation fulfilled all three Coronary Vasomotion Disorders International Study Group (COVADIS) diagnostic criteria for definitive epicardial CAS: (i) > 90% vasoconstriction (complete occlusion), (ii) reproduction of typical angina symptoms, and (iii) ischaemic ECG changes.^10^ Formal acetylcholine challenge was therefore deemed unnecessary. Following intracoronary glyceryl trinitrate (350 µg), the LAD regained normal calibre and TIMI III flow (see Supplementary material online, *Video S3B*). Further, comprehensive microvascular characterization demonstrated normal coronary flow reserve (CFR, 3.2) and index of microcirculatory resistance (IMR, 5), confirming the absence of coronary microvascular dysfunction (*Figure 4*).

As the diagnosis of CAS was confirmed, metoprolol, which was initiated as part of standard post-cardiac arrest care, was promptly discontinued.^7,9^ Diltiazem modified release (MR) was introduced at 180 mg once daily and uptitrated to 360 mg over 4 weeks in the outpatient setting. In keeping with the ESC Class IIa (Level C) recommendation for ICD in sudden cardiac arrest survivors with CAS,^11^ a secondary prevention ICD including the subcutaneous ICD (S-ICD) was discussed; however, the patient declined following shared decision-making. Lifestyle modifications such as vaping cessation were advised and atorvastatin 20 mg was initiated for mild mid-LAD atherosclerosis. At 12 months, the patient remains extremely well with no recurrence of angina, arrhythmia, or syncope and has returned to regular exercise. However, declining ICD implantation precluded return to work as a crane operator.

## Discussion

CAS-related cardiac arrest is under-diagnosed but potentially lethal, accounting for 2–7% of all cases of OOHCA.^4,5^ Timely diagnosis enables institution of endotype-specific therapy with enhanced clinical outcomes.^5^

Pathophysiologically, CAS involves transient epicardial vasoconstriction driven by vascular smooth muscle hyperreactivity and endothelial dysfunction, triggered by smoking, catecholamine surges, and cold exposure.^12^ The COVADIS criteria standardizes the invasive diagnosis, and our patient met all three through spontaneous spasm alone.^10^ Takagi *et al*. reported that CAS survivors of OOHCA had significantly worse 5-year outcomes compared with non-arrest CAS patients,^5^ and Ahn *et al*. demonstrated a cardiac death rate of 24.1 per 1000 patient-years in aborted sudden cardiac death due to CAS.^13^ Our patient exhibited multiple high-risk features: (i) male sex, (ii) spontaneous LAD occlusion, (iii) VF arrest, and (iv) ST elevation.^5,12,14^ The COVADIS group provides a Class I recommendation to perform CFT in unexplained resuscitated cardiac arrest,^10^ and the current Japanese Circulation Society (JCS) 2023 guidelines provide a Class IIb recommendation for acetylcholine provocation in MINOCA, which is further reflected in the American Heart Association (AHA) scientific statement.^7,8^

An important consideration is whether post-ROSC ST elevation could reflect defibrillation-related ECG changes. However, the troponin elevation from 4 to 77 ng/L confirmed acute myocardial injury; occurrence of anterior ST elevation with spontaneous CAS during CFT established a clear mechanistic link; and comprehensive evaluation excluded a primary electrical or structural heart disease. Furthermore, CMRi, whilst negative for late gadolinium enhancement, does not exclude transient ischaemia without established necrosis. Thus, MINOCA should be regarded as a working diagnosis mandating systematic investigation.^3,6^

A cornerstone of CAS management includes non-dihydropyridine calcium channel blockers, long-acting nitrates and nicorandil.^7,9,12^ Beta-blockers should be avoided given the risk of exacerbating vasospasm.^7,9^ Lifestyle interventions including nicotine cessation should be implemented.^7,9^

Although CAS leading to cardiac arrest is a potentially reversible event, observational data demonstrate a 3–6% annual recurrence rate of life-threatening ventricular arrhythmias despite optimal medical therapy.^4,13^ Consequently, the 2022 ESC guidelines provide a Class IIa (Level C) recommendation for ICD in sudden cardiac arrest survivors with CAS.^11^ The JCS 2023 guidelines adopt a two-tiered approach: Class IIa when medical therapy is ineffective, and Class IIb when effective.^7^ The S-ICD may be particularly suited to this population, as CAS-related arrhythmias are typically polymorphic VT/VF, obviating the need for anti-tachycardia pacing.

## Conclusion

Cardiac arrest secondary to CAS remains under-recognized and an important therapeutic target. CFT should be considered in patients with unexplained resuscitated cardiac arrest and initiation of endotype specific therapy may enhance clinical outcomes. Yet, those who have suffered cardiac arrest represent a higher risk cohort and ICD therapy should be considered following shared decision-making.

## Lead author biography


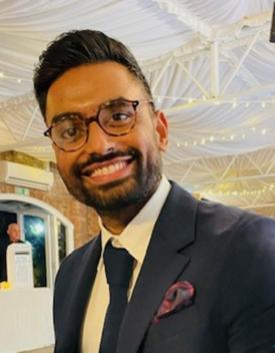


Dr Aswin Babu is a cardiology registrar and PhD candidate at the University of Western Australia and Royal Perth Hospital. His clinical and research interests include acute coronary syndromes, coronary microvascular dysfunction, and invasive coronary physiology.

## Supplementary Material

ytag442_Supplementary_Data

## Data Availability

The data underlying this article are available in the article itself. Any further information will be shared on reasonable request to the corresponding author.
